# Neurophysiological Synchrony Between Children With Severe Physical Disabilities and Their Parents During Music Therapy

**DOI:** 10.3389/fnins.2021.531915

**Published:** 2021-04-30

**Authors:** Ali Samadani, Song Kim, Jae Moon, Kyurim Kang, Tom Chau

**Affiliations:** ^1^Philips, Cambridge, MA, United States; ^2^School of Optometry & Vision Science, University of Waterloo, Waterloo, ON, Canada; ^3^Holland Bloorview Kids Rehabilitation Hospital, Toronto, ON, Canada; ^4^Institute of Biomedical Engineering (BME), University of Toronto, Toronto, ON, Canada; ^5^Music and Health Science Research Collaboratory (MaHRC), Faculty of Music, University of Toronto, Toronto, ON, Canada

**Keywords:** neural synchrony, spectral coherence, granger influence, child-parent dyad, interbrain synchrony, EEG, children with severe physical disabilities, music therapy

## Abstract

Although physiological synchronization has been associated with the level of empathy in emotionally meaningful relationships, little is known about the interbrain synchrony between non-speaking children with severe disabilities and their familial caregivers. In a repeated measures observational study, we ascertained the degree of interbrain synchrony during music therapy in 10 child-parent dyads, where the children were non-speaking and living with severe motor impairments. Interbrain synchrony was quantified via measurements of spectral coherence and Granger causality between child and parent electroencephalographic (EEG) signals collected during ten 15-min music therapy sessions per dyad, where parents were present as non-participating, covert observers. Using cluster-based permutation tests, we found significant child-parent interbrain synchrony, manifesting most prominently across dyads in frontal brain regions within *β* and low *γ* frequencies. Specifically, significant dyadic coherence was observed contra-laterally, between child frontal right and parental frontal left regions at *β* and lower *γ* bands in empathy-related brain areas. Furthermore, significant Granger influences were detected bidirectionally (from child to parent and vice versa) in the same frequency bands. In all dyads, significant increases in session-specific coherence and Granger influences were observed over the time course of a music therapy session. The observed interbrain synchrony suggests a cognitive-emotional coupling during music therapy between child and parent that is responsive to change. These findings encourage further study of the socio-empathic capacity and interpersonal relationships formed between caregivers and non-speaking children with severe physical impairments.

## 1. Introduction

### 1.1. Interpersonal Physiological Synchrony

Interpersonal synchronization broadly describes the spontaneous, contemporaneous alignment of physiological indicators or the automatic mirroring of behaviors between people during social interactions (Feldman, 2007). Behavioral synchronization has been observed in many interpersonal activities, including co-ambulation (Bernieri and Rosenthal, 1991) and rhythmic movements of child and therapist during music therapy (Dvir et al., 2020). Physiological synchrony may involve the autonomic nervous system, as in the respiratory alignment in psychotherapist-client dyads (Tschacher and Meier, 2020), or the central nervous system, as in hemodynamic synchronization in the prefrontal cortex of the brain during cooperative game play (Liu et al., 2016). In fact, behavioral and physiological synchrony can co-occur, as recently clarified by Gordon et al. (2020) in their study of the covariation of behavioral and autonomic nervous system synchrony during group drumming tasks.

In the mutually calibrated physiological state, people are said to be in “sync” with each other. The strength of such synchrony can be quantified through a plethora of measures, including for example, cardiac rhythms between mothers and infants (Feldman et al., 2011), cortisol readings between mothers and adolescents (Papp et al., 2009), electrodermal activity between patients and therapists (Marci et al., 2007), and hemodynamic brain responses (i.e., higher-order information and emotion processing) between speakers and listeners (Stephens et al., 2010).

### 1.2. A Putative Link Between Synchrony and Socio-Emotional Connection

When an individual observes or interacts with another human being, there is a tendency to adopt the physiology and behaviors corresponding to the partner's affective state (Stephens et al., 2010; Ebisch et al., 2012). This unconscious calibration of one's own body and brain to another's emotional experience has been associated with motor, cognitive, and emotional empathy. For example, Pauly et al. (2020) observed that stronger cortisol synchrony occurred in the presence of a partner and subsequent to positive socio-emotional interactions in older couples. Likewise, Coutinho et al. (2019) found greater electrodermal synchrony during positive interactions between romantic couples when the males had higher dyadic empathy. This connection between synchrony and socio-emotional experience is not limited to the peripheral nervous system and extends to the brain. For example, Dikker et al. (2017) reported that empathy predicted the amount of electroencephalographic brain synchrony among high school students in a naturalistic classroom setting. More recently, Azhari et al. (2020) showed that the unique presence of a spouse elevated a couple's hemodynamic brain-to-brain synchrony while attending to salient stimuli.

While the above evidence points to an association between synchrony and socio-emotional bonding, the putative role of mirror neurons in this association remains contested (Hickok, 2009; Lamm and Majdandžić, 2015). Nonetheless, it has been suggested that empathy has neurobiological underpinnings in the mirror neuron system (Gallese, 2001; Tononi, 2020) or more broadly the action-observation network (Jospe et al., 2020), the constellation of neurons that allows one to anticipate the goals of others through observation of their associated actions.

### 1.3. Music and Interpersonal Synchrony

There is growing evidence that music affords a facilitative environment for the development of both behavioral and physiological synchrony. When an experimenter tapped synchronously to music with a participant, the latter behaved in a more helpful manner toward the former (Stupacher et al., 2017). The same was not observed when tapping asynchronously or to a metronome. In a joint-music making task requiring no previous musical training, Novembre et al. (2019) found that participants with high levels of empathy were more accurate at synchronizing their musical output with that of another person. Indeed, literature has suggested that synchronized rhythmic behavior in collective settings such as dance and music promotes group cohesion and cooperative behavior (Freeman, 1998; Bispham, 2009).

On the physiological front, Gordon et al. (2020) reported increased synchrony of cardiac interbeat intervals of participants during a synchronous group drumming task, while Ardizzi et al. (2020) noted elevated levels of cardiac synchrony in an audience watching a live performance together, contending that a collective aesthetic experience may enhance group cohesion. Of particular relevance to the present study, (Fachner et al., 2019) reported that peaks in frontal and parietal alpha asymmetries in electroencephalographic recordings of therapist and patient aligned at various points during music therapy. Recently, using an EEG hyperscanning method, (Fachner et al., 2019) reported that classical music induced meaningful interbrain synchronization between a music therapist and a client during sessions of Guided Imagery and Music.

Music can evoke emotions or modulate ongoing emotions expressed through physiological arousal (automatic and endocrine changes) and motoric expression (e.g., smiling, clapping, dancing, and singing) (Koelsch, 2014). Musical communication also facilitates social, emotional, and cognitive development for infants and young children (Trehub, 2003; Fitch, 2006).

### 1.4. The Importance of Parent-Child Brain-to-Brain Synchrony

Brain-to-brain synchrony between parent and child is an emerging neurobiological marker of socio-emotional development. For example, Reindl et al. (2018) recently observed interbrain synchrony only in a parent-child cooperative play condition, whereas child-stranger play, whether cooperative or competitive, yielded no such synchrony. This finding suggests that synchrony reflected the unique emotional bond between parent and child, which the authors note is related to the child's development of emotion regulation. Others have associated parent-child synchrony with positive social interactions (Cui et al., 2012), development of empathy during childhood and adolescence (Feldman, 2007), cognitive processing in infancy and school adjustment (Leclère et al., 2014), and the development of self-regulation such as recovery from periods of irritability (Quiñones-Camacho et al., 2020). By extension, the emerging importance of synchrony as a neurophysiological predictor of a broad range of developmental outcomes suggests the eventual need to study the risks to parent-child synchrony and the interventions which may strengthen such interbrain coupling.

### 1.5. Propensity for Interbrain Synchrony in Non-speaking Children With Severe Physical Disabilities

Few studies have quantified the development of interbrain synchrony in children with severe disabilities and limited or delayed expressive communication. For example, Kasari et al. (2003) reported that children with Down syndrome exhibited more prosocial behaviors than typically developing peers in response to distress in others, but no corresponding study of interbrain synchrony involving this population has yet been published. In terms of the functional brain, atypical mirror neuron system activation has been observed in children with cerebral palsy, compared to their typically developing counterparts (Errante et al., 2019). Anatomically, reductions in gray matter volume of the salience and mirror neuron networks have been reported in pediatric brain injury and have been associated with lower Theory of Mind scores, suggesting social-cognitive impairment (Ryan et al., 2017). Further, it is well-documented that children with the most severe forms of cerebral palsy have the most restrictions in social function and communication (Voorman et al., 2010). Taken collectively, these studies and others point to likely impacts on the propensity for interbrain synchrony among non-speaking children with severe physical disabilities but the degree and nature of such impacts remain largely unexplored.

The above literature supports the notion that synchrony plays an important role in interpersonal interactions, that music has potential to serve as a facilitator of synchrony, and that little is known at present about interbrain synchrony in non-speaking children with severe disabilities. In the local health system, music therapy is a service afforded to children and youth with severe physical disabilities. However, to date, very little is known about the capacity for synchronization between a non-speaking child with severe physical disabilities and cognate caregivers given the limited opportunities for conventional interpersonal interaction through conversational speech and transactional gestures. The measurement of neurophysiological synchrony in a music therapy context may thus shed light on the social connections between children with severe disabilities and parental caregivers. In turn, this information may inform the provision of optimal and personalized opportunities for development.

The present study sought to answer the following questions.

1. Does parent-child brain synchrony (as measured by coherence and Granger influence) during music therapy exceed levels observed during corresponding pre-session baselines?
2. Does parent-child brain synchrony (as measured above) increase over successive time intervals within a music therapy session?

Based on the reviewed literature, we expected the presence of neural synchrony between parent and child during music therapy at levels significantly above baseline. We further hypothesized that the level of synchrony would increase throughout the music therapy session.

## 2. Materials and Methods

### 2.1. Participants

We recruited a convenience sample of 10 participants with disabilities (14.7 ± 6.7 years, 7 males, 3 females), one of their parents (7 mothers; 3 fathers), and one music therapist (female) from a local pediatric rehabilitation hospital. Participants were not actively taking seizure medications or other medications that affect heart rate, cortisol levels or skin conductance. The child/youth participants were all non-speaking and had one or more severe forms of disability, including cerebral palsy, seizure, epilepsy, and anoxic brain disorder. All child/youth participants had profound physical limitations (e.g., children with cerebral palsy were all level V on the Gross Motor Classification System). Table 1 lists the age, sex and residential status of the child/youth participants. Parents or therapists with a history of neurological, cardiopulmonary, or metabolic illness or with current medication affecting the peripheral nervous system were excluded. All participants were asked to refrain from eating (2 h prior), drinking caffeine (4 h prior), and smoking (4 h prior). All participants were compensated for their time. The therapist and the parents, both as participants and proxy decision makers for their children, gave written informed consent. The Research Ethics Boards of the Holland Bloorview Kids Rehabilitation Hospital and University of Toronto approved the study protocol.

**Table 1:** Child/youth participant characteristics.

**Child/youth participant**	**Age**	**Sex**	**Residential status**
1	26	F	Inpatient
2	20	M	Outpatient
3	19	M	Outpatient
4	15	M	Inpatient
5	9	M	Inpatient
6	3.5	M	Outpatient
7	18	F	Inpatient
8	7	F	Inpatient
9	15	M	Outpatient
10	15	M	Outpatient

### 2.2. Experimental Design

**Figure 1:**
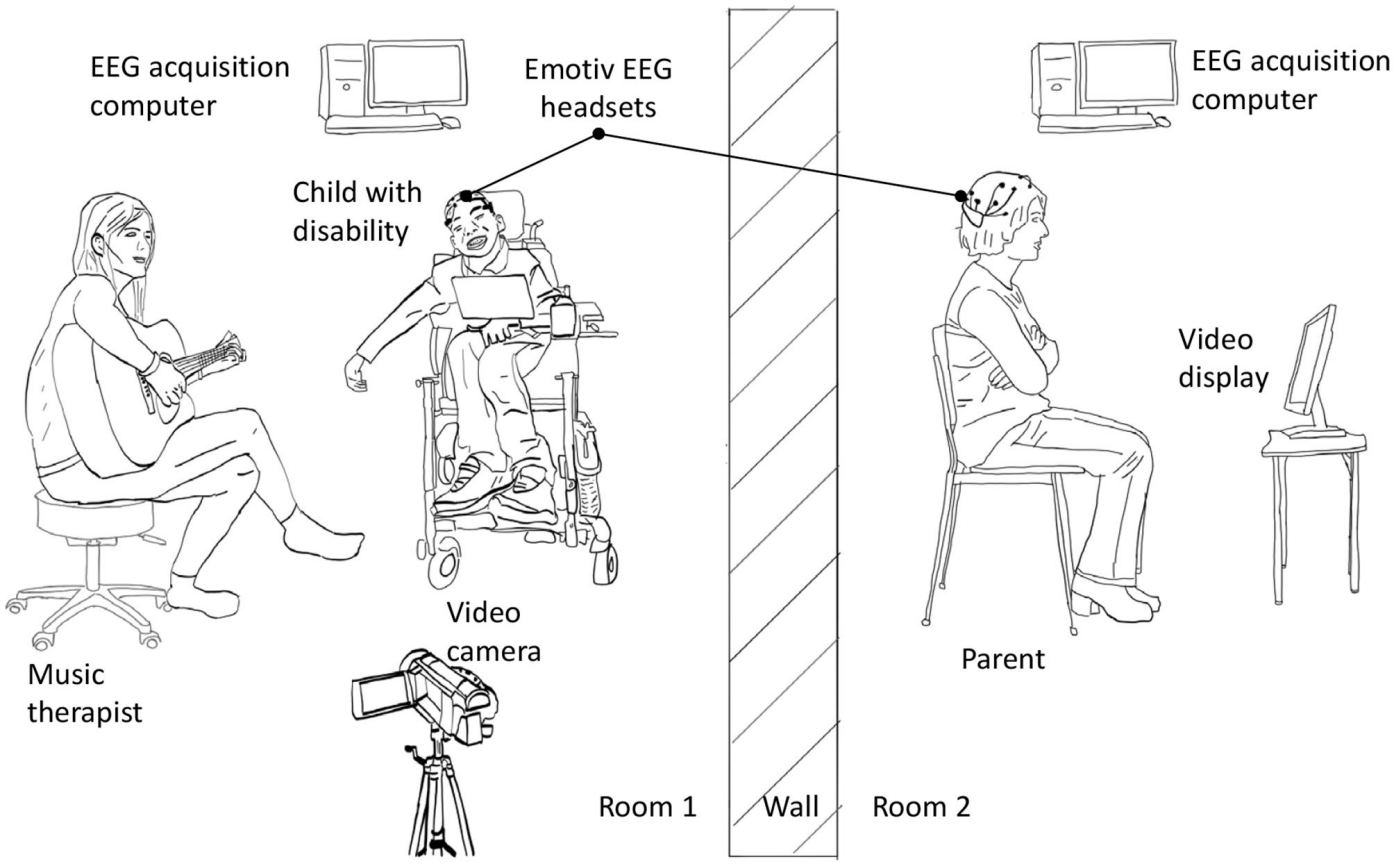
Experimental setup for simultaneous EEG recording from children and their parents. For clarity, wired connections between data acquisition computer and sensors are not shown.

**Figure 2:**
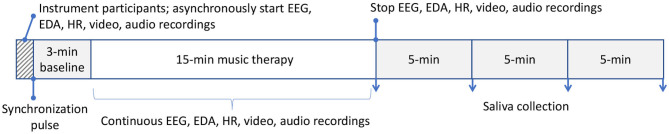
Sessional protocol. All data streams were synchronized with an external sync pulse. Saliva was collected post-session. Only EEG data are reported here. Each of the 10 sessions per participant followed the same protocol. EEG, electroencephalography; EDA, electrodermal activity; HR, heart rate.

Each child-parent dyad attended ten 18−min experimental sessions that were no more than two weeks apart. Each session consisted of a 3-min baseline followed by a 15−min music therapy session. All participants sat quietly without any interactions or music during the 3-min baseline period. The parents watched and listened to the therapy sessions via a live audio-video feed in either a separate room or in a partitioned area so that there were no interactions with their children or the therapist. The camera was positioned such that the parents had exclusively a frontal view of the faces of their children. This design was adopted to emulate current clinical practice. Both rooms were maintained at 20–23 °C and 35% relative humidity. EEG signals were simultaneously recorded from participants (except therapist) with the wireless Emotiv EPOC^1^ headsets throughout the baseline and therapy session at 128 Hz. The EEG headset consisted of 14 channels permanently situated at AF3, AF4, F7, F8, F3, F4, FC5, FC6, T7, T8, P7, P8, O1, and O2 according to the International 10−20 system. The headset deployed saline-based electrodes. The child and parental EEG signals were recorded on separate computers. Experimental recordings were synchronized temporally via a button press that annotated all data streams (including video and audio) with a common sync pulse. Custom software and hardware were developed for this purpose. Additionally, electrodermal activity and heart rate were collected continuously throughout baseline and the therapy session. Saliva was collected four times from parent and child after the session in 5-min intervals. Figure 1 summarizes the experimental setup while Figure 2 depicts the sessional protocol. Only the EEG data are presented in this paper.

### 2.3. Music Therapy

Music and instrument selection were customized to the preference of each child and documented according to standardized guidelines for reporting music-based interventions (Robb et al., 2011) in Tables 2–4, which summarize the intervention theory, sample content and example implementation, respectively. The list of each participant's favorite songs was assembled through conversations with the attending parent prior to the commencement of the study sessions. Familiar music tends to recruit activity in neural structures involved socio-emotional responses (Freitas et al., 2018; Wallmark et al., 2018).

**Table 2:** Intervention theory.

**Rationale for music selection** Preferred music according to parental recommendation and documentation of music by previous music therapists who had worked with the child (if applicable); further selections of songs similar in genre, artist and style to those familiar to child; improvisation introduced at discretion
**Accompanying instruments:**
- Hand Sonic 10 electric drum (child 8)
- Piano (child 12)
- Violin (child 7)
- Small djembe drum (child 9)
- Wind chimes (child 2)
- Cymbals (child 4)
- Wrist bells, Cabassa, Mallets
^*^ Some instruments were selected more frequently for certain children as shown in brackets
**Expected impact:** Encourage engagement, alertness/wakefulness, and music participation

**Table 3:** Sample intervention content.

**Instrument**	**Musical piece**
Piano	“Ave Maria” (Franz Schubert)
	“A Whole New World” (Alan Menken)
	“Banana Phone” (Raffi)
	“Feliz Navidad” (Jose Feliciano)
	“I Got Rhythm” (George Gershwin)
	“I Have a Dream (ABBA)”
	“Lean on Me” (Bill Withers)
	“Oh Canada”
	“White Christmas” (Irving Berlin)
	“Yankee Doodle”
Guitar	“Baby Beluga” (Raffi)
	“Black Hole Sun” (Soundgarden)
	“Down by the Bay”
	“Something in the Way” (Nirvana)
	“Stand by Me” (Ben E. King)
	“Sweet Home Alabama” (Lynyrd Skynyrd)
	“The Lion Sleeps Tonight (George David Weiss, Hugo Peretti, and Luigi Creatore)”
	“Where Did You Sleep Last Night” (anonymous; as performed by Nirvana)
	“Yellow (Coldplay)
	“Yellow Submarine” (Raffi)
	“Yellow Submarine” (The Beatles)
	“You are My Sunshine”
	“3 AM” (Matchbox 20)
	“Fallout” (Marianas Trench)
	“Good Bye Song” (therapist)
	“Have You Ever Seen the Rain?” (John Fogerty; as performed by Credence Clearwater Revival)
	“Hey Jude” (The Beatles)
	“Last Kiss” (W. Cochran et al.; as performed by Pearl Jam)
	“Let it Be” (The Beatles)
	“Mr. Sun” (Raffi)
	“Never Too Late” (Three Days Grace)
	“Ob-la-di Ob-la-da” (The Beatles)
	“Oh Susanna” (Stephen Foster)
	“Over the Rainbow” (E.Y. Harburg (lyrics) and Harold Arlen (music))
	“Proud Mary” (John Fogerty; as performed by Credence Clearwater Revival)
Guitar + electric drum	“La Bamba” (Mexican folk song; written by Ritchie Valens)
Violin	“Por una Cabeza” (Argentine Tango; Carlos Gardel)
	“Salut d'Amour” (Edward Elgar)
Drum	“Kumbaya”, “The Ants Go Marching One by One”, “When the Saints Go Marching In” (anonymous)
	“Fungalafia”

**Table 4:** Intervention checklist.

Music delivery method	Interventionist only; one-on-one; live
Intervention materials	Therapist's voice (for every musical excerpt) and instruments mentioned above. No non-music materials were used.
Intervention strategies	Incorporation of child's name and vowel sounds into therapist's vocalizations through improvised music
	Various percussion instruments as accompaniment
	Use of dynamics to match child's apparent alertness
	Variation in rhythm, tempo, pitch and dynamics often applied to promote engagement and alertness
	Mirroring and mimicking of child motion or vocalization to promote engagement
Intervention delivery schedule	Ten 15-min sessions (except for child 1: six 15-min sessions); no more than 2 weeks apart
Interventionist	Qualified music therapist at Holland Bloorview.)
Treatment fidelity	Clinician had a Certified Music Therapist (MTA) designation from the Canadian Association for Music Therapists, which indicates they have met the guidelines and standards for professional competence set out by the Association.
Setting	Complex Continuing Care Activity Room or Music Therapy Room at Holland Bloorview; private; ambient sound was negligible
Unit of delivery	One-on-one

Only the music therapist played an instrument although an instrument might be placed in the hand, on the lap, or in proximity of a child to encourage participation. Each child or youth participant sat in their wheelchair while the music therapist faced them, either in a seated or standing posture, such that eye contact could be encouraged. The music therapist communicated with each pediatric participant through improvised lyrics, sung to the melody of familiar songs selected by the participant. For example, the music therapist might have sung, "Lisa plays the drum, drum, drum" when the participant may have gestured to a drum positioned near them. The music therapist also sang about the participant's actions or responses and how she wanted the child to participate. Particularly noteworthy are the intervention strategies deployed by the attending music therapist to promote engagement and alertness (Table 4), such as strategic incorporation of the child's name in the music, on-the-fly modulations of rhythm, tempo, pitch, and dynamics to match the child's level of alertness and mirroring of the child's behavior.

The iso principle (Altshuler, 1948) broadly prescribes the matching of musical components (e.g., rhythm, tempo, pitch, and dynamics) to the patient's mood or physiological states (Davis et al., 2008) and has been widely applied in music therapy to build therapeutic relationship, connect patients with music, and gradually shift the patient's mood or physiological state (Heiderscheit and Madson, 2015; Kim et al., 2018). This principle can guide musical improvisation to maximize the effectiveness of music therapy for children with disabilities (Salomon-Gimmon and Elefant, 2019; Stegemann et al., 2019).

In this study, the music therapist improvised using written music by changing the words and extending the original songs. The therapist also improvised using ostinato patterns as well as altered melodies and chords. However, the improvisation was constrained within the personalized music list of each participant. Overall, the music therapist deployed a combination of free improvisation and written music. The music intervention was adapted to meet the specific participation level, physical condition, and cognitive skills of each individual participant. The music therapist was trained in neurologic music therapy (Thaut and Hoemberg, 2014) and thus deployed techniques thereof, as appropriate.

### 2.4. Data Analysis

Prior to signal analyses, all data streams were aligned at the synchronization pulse. Raw EEG signals during a 15−min music therapy session were divided into three sub-intervals of 5-min each, denoted as MT1, MT2, and MT3. These sub-intervals along with the preceding 3-min baseline EEG recordings were segmented into overlapping windows of 1-s duration (referred to as a trial, hereafter) with a 90% overlap. Spectral densities of the EEG trials from child and parent were then computed using fast Fourier transform with 4 Hz spectral smoothing through multi-tapering (McCoy et al., 1998) using 3 Slepian (discrete prolate spheroidal) sequences to suppress temporal jitters. The resulting spectral densities were in turn used to compute spectral coherence and Granger influences between child-parent dyads. Spectral densities, coherence, and Granger influences were computed using FieldTrip (Oostenveld et al., 2011). Coherence quantifies the frequency-specific covariation between two signals and is given by the magnitude squared of their cross-spectral density over the product of their individual spectral densities (Bendat and Piersol, 2011). Granger influence captures the directional influence of one time series on another, i.e., the degree to which future values of one time series are predicted by the combination of lagged values of itself plus those of a second time series (Granger, 1969). Child-parent spectral coherence and directional Granger influences between EEG channel-pairs were computed for baseline and music therapy subintervals (MT1, MT2, and MT3). Connectivity measures (coherence and Granger influence) were computed for every 20-s recording (188 overlapping trials) with a 2−s shift between subsequent 20-s windows. This resulted in 144 and 251 connectivity measures for the baseline and each one of the music therapy subintervals, respectively. To determine whether the connectivity measures during the music therapy subintervals were significantly higher than that of the corresponding baseline, the Wilcoxon rank sum test was performed. Furthermore, connectivity measures between successive music therapy subintervals (i.e., baseline vs. MT1; MT1 vs. MT2 and MT2 vs. MT3) were compared using the Wilcoxon rank sum test to identify significant changes in connectivity over the course of the music therapy session.

**Figure 3:**
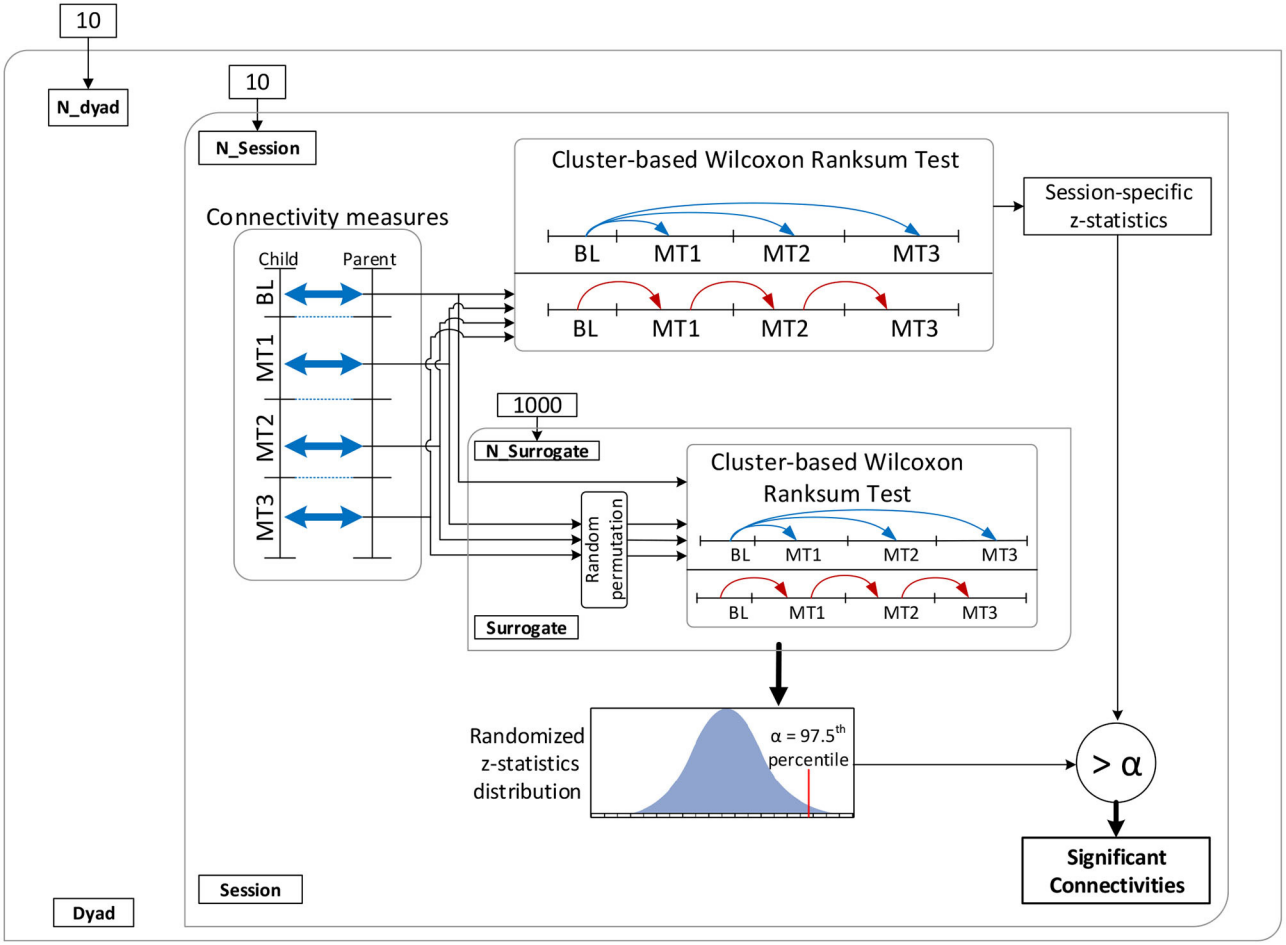
Schematic of the connectivity analysis. BL, baseline; MT1, first 5 min of music therapy; MT2, second 5 min of music therapy; MT3, last 5 min of music therapy.

**Figure 4:**
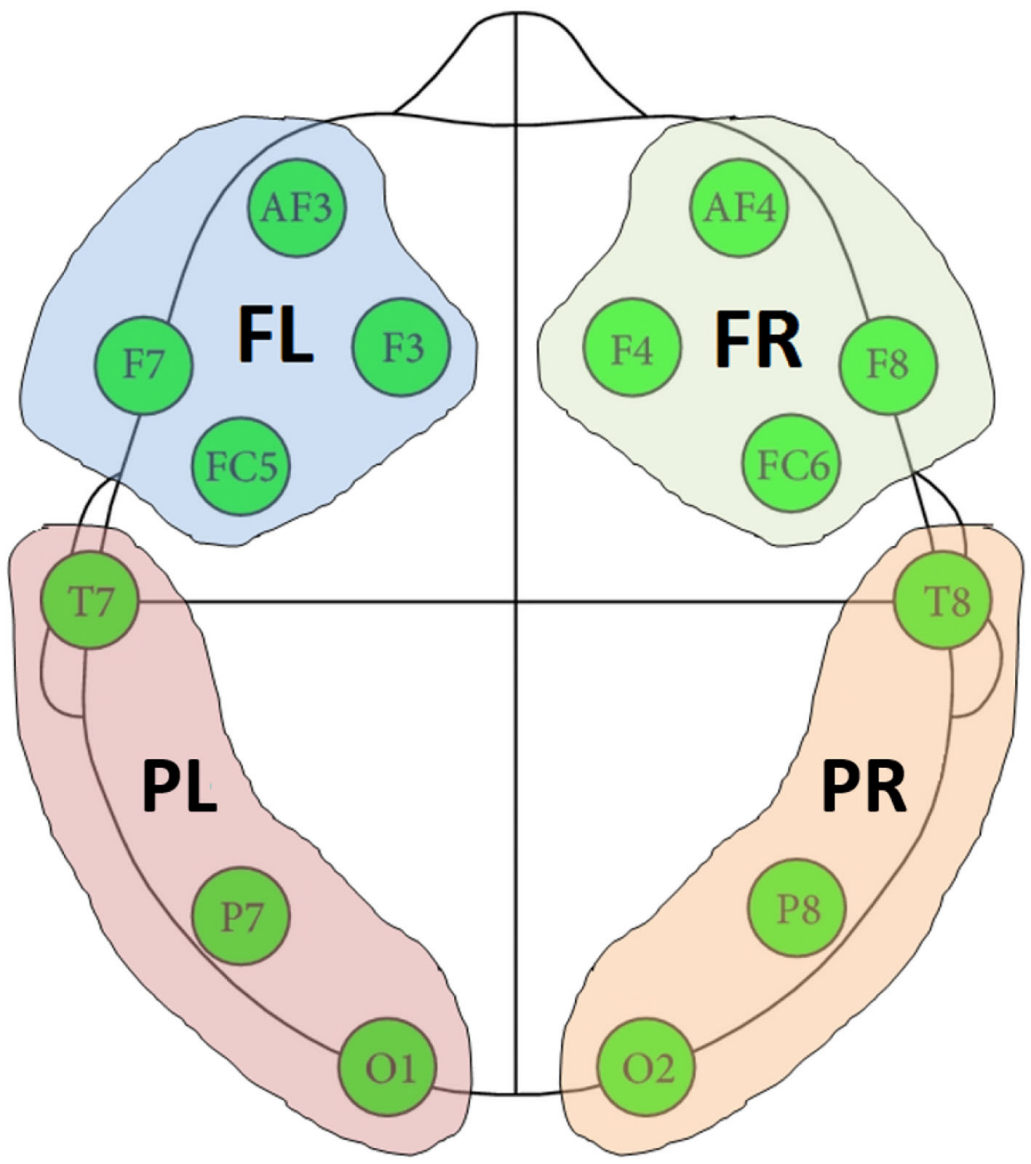
Four regions formed based on the location of EEG channels. FR, frontal right; FL, frontal left; PR, posterior right; PL, posterior left.

A cluster-based permutation test (Groppe et al., 2011a) was implemented to evaluate the above hypotheses about connectivity changes. Cluster-based approaches are touted as the most sensitive univariate mass test for broadly distributed effects (Groppe et al., 2011b). Figure 3 shows the implemented dyadic connectivity analysis. A rank sum test was performed for connectivity measures computed for every frequency bin (1 to 64 Hz) and parent-child × channel-pairs. Only cases with *p* < 0.05 and at least a medium Cohen's d effect size (*d* > 0.5) were retained. For the cluster-based permutation test, clusters were formed based on 5 frequency bands and 4 brain regions (Figure 4). The frequency bands were in the *δ* band (0–4 Hz), *θ* band (4–8 Hz), *α* (8–14 Hz), *β* band (14–32 Hz), and lower *γ* band (32–64 Hz). Among these bands, the suppression of *α* band power and increased lower *γ* band activity are associated with shared brain activations between people, attention, perceptual awareness, and cognitive control (Fries, 2005; Wyart and Tallon-Baudry, 2008; Frenkel-Toledo et al., 2014) which may subserve empathy. The cluster-based analysis resulted in 16 child-parent region-pairs for each frequency band. Clusters where the number of active members (*p* < 0.05 and medium to large Cohen's d) constituted less than 10% of the cluster size were ignored. The z-statistics of a cluster were summed up to produce cluster-level z-statistics (“mass of the cluster”). For the permutation runs, 1,000 permutations were used where in each permutation, the order of connectivity measures during the entire music therapy recording (15−min) was randomized. The baseline connectivities remained unchanged during the permutation test. Cluster masses were computed for the 1,000 randomized permutations as described above.

Subsequently, a connectivity value was identified as significant at *p* < 0.05 if its z-statistic was higher than the 97.5 th percentile of the cluster-based permutation z-statistics, after correcting for multiple comparisons across 16 brain-region pairs and 5 frequency bands. The multiple comparison correction was done by comparing the rank sum z-statistics against the maximum 97.5 th percentile of the randomized z-statistics for 5 (frequency bands) × 16 (brain regions) clusters (Bosman et al., 2012).

*Post-hoc* analysis (a separate linear mixed-effect regression model) was performed to examine the effects of child sex and residential status (inpatient, outpatient), along with brain region and frequency bands, on coherence and Granger influences (dependent variables). Additionally for Granger values, the effect of the direction of analysis (i.e., parent to child or child to parent) was also tested. While sex, residential status, brain region, frequency band and directionality were included as fixed effects, subjects, sessions, and child age were represented in the model as random effects. Type III Wald F tests with Kenward Roger degrees-of-freedom approximation were used for testing the fixed effects. SPSS statistical package was used to run the *post-hoc* analysis.

## 3. Results

**Figure 5:**
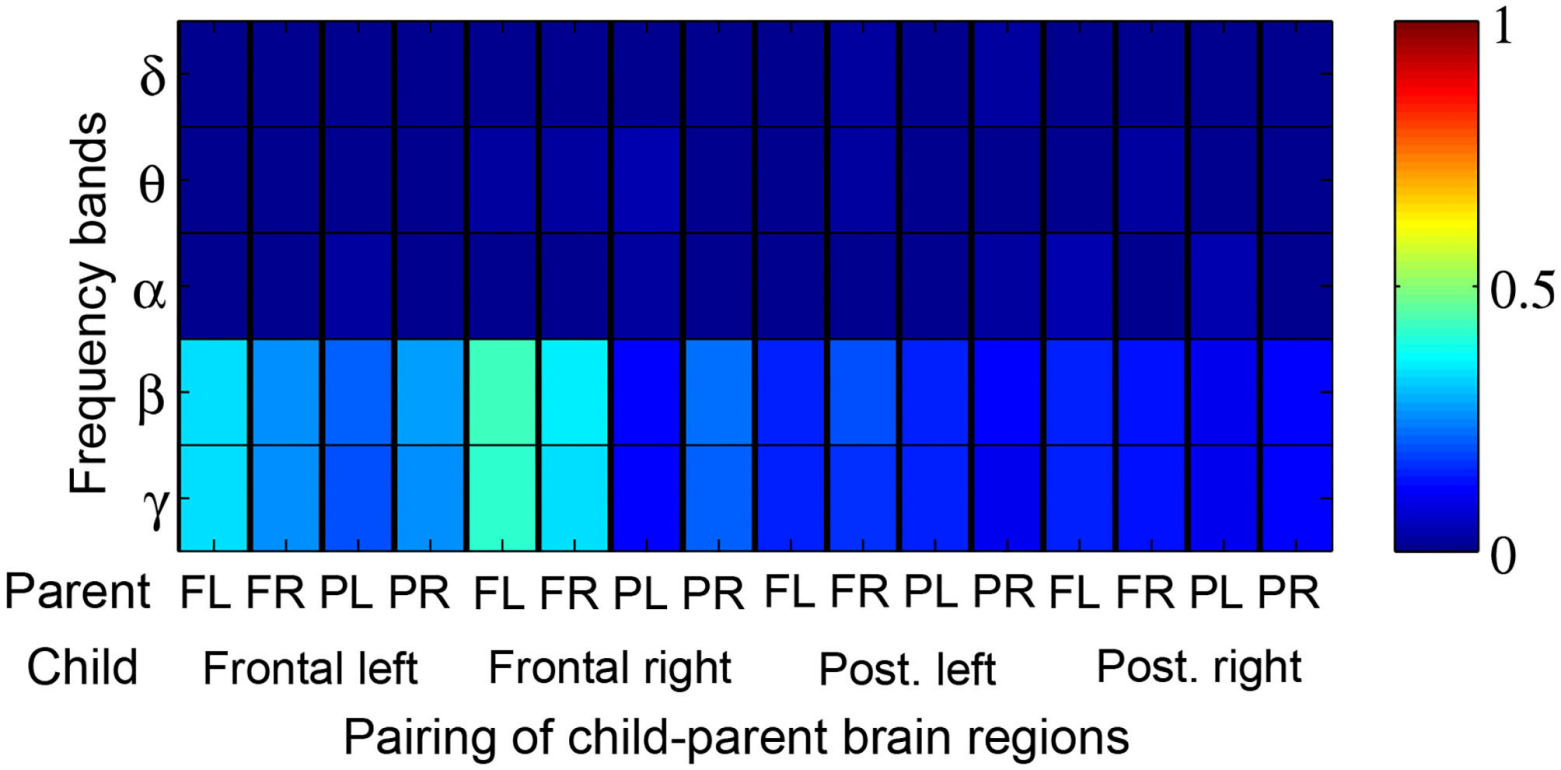
Proportion of child-parent dyads exhibiting significant music therapy coherences with respect to their corresponding baseline segments. Red shading thus denotes 100% of dyads with significant coherence. The proportions are tabulated by frequency band (rows) and pairings of child-parent brain regions i.e., frontal left (FL), frontal right (FR), posterior left (PL), and posterior right (PR).

**Figure 6:**
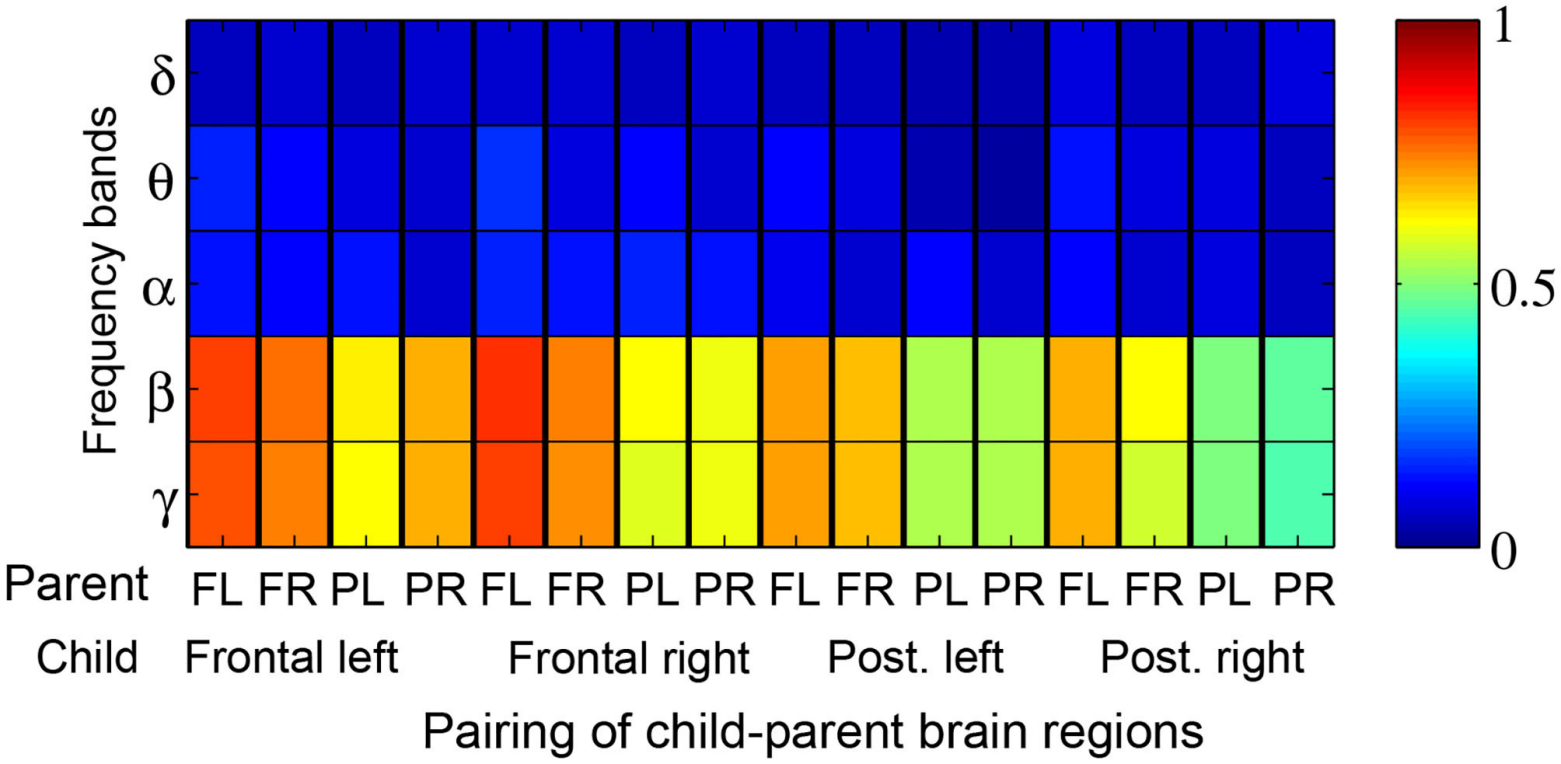
Proportion of child-parent dyads exhibiting significant increases in coherence over the course of a music therapy session. Red shading thus denotes 100% of dyads with significant increases in coherence. The proportions are tabulated by frequency band (rows) and pairing of child-parent brain regions i.e., frontal left (FL), frontal right (FR), posterior left (PL), and posterior right (PR).

Figure 5 depicts the proportion of child-parent dyads who exhibited significant inter-brain coherence during music therapy sessions, indexed by frequency band (vertical axis) and pairing of brain quadrants (horizontal axis). In this figure, a red shaded square means that all the child-parent dyads had significant coherence at the given frequency band and pairing of brain regions. Significant coherences were found to occur most often in the *β* and lower *γ* bands, between child frontal right and parental frontal left brain regions. Over successive sub-intervals of music therapy sessions, a significant increase in coherence over time (Figure 6) was observed in *β* and lower *γ* bands in almost all dyads, again between the child's frontal right and parental frontal left brain regions. Frontal left brain regions of parent and child also showed significant increases in coherence over the session for the majority of dyads.

**Figure 7:**
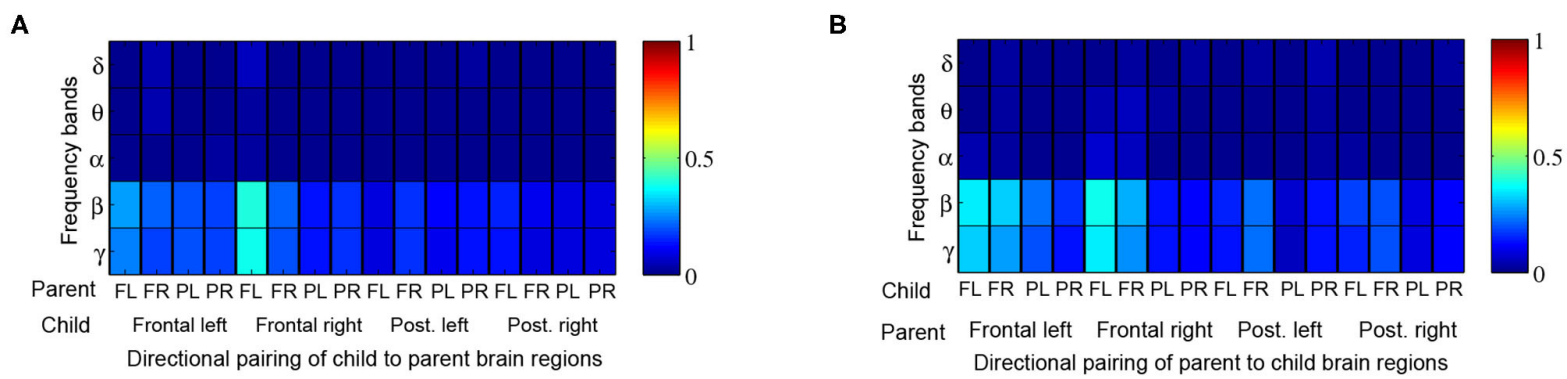
Proportion of child → parent and parent → child directional analyses exhibiting significant Granger influences during music therapy compared to preceding baseline values. Red shading thus denotes 100% of dyads with significant Granger influence. The proportions are tabulated by frequency band (rows) and directional pairing of brain regions, i.e., frontal left (FL), frontal right (FR), posterior left (PL) and posterior right (PR) from **(A)** child to parent, and **(B)** parent to child.

**Figure 8:**
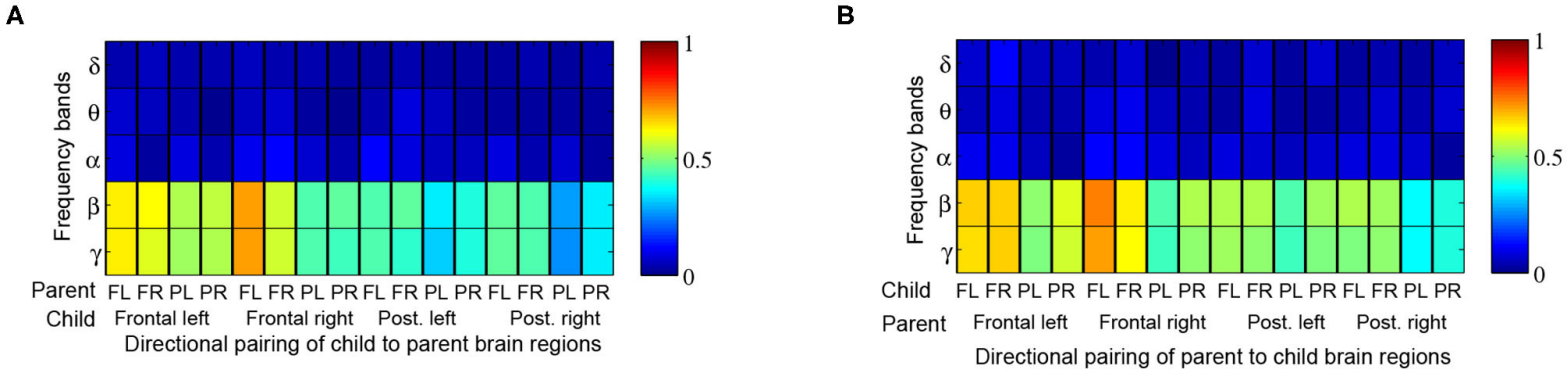
Proportion of child → parent and parent → child directional analyses exhibiting significant increases in Granger influences over the course of a music therapy session. The proportions are tabulated by frequency band (rows) and brain region, i.e., frontal left (FL), frontal right (FR), posterior left (PL), and posterior right (PR) from **(A)** child to parent, and **(B)** parent to child.

When comparing Granger influences (i.e., direction and magnitude of influence from child to parent and vice versa) during music therapy sessions with those of corresponding baselines, no prominent pattern was observed (Figure 7). Granger influences, however, significantly increased over the course of the music therapy session bidirectionally between child and parent, as indicated in Figures 8A,B. Similar to coherence measures, significant Granger influences between the oscillations deriving from the child's frontal right and parent's frontal left brain regions in the *β* and lower *γ* frequency bands were the most frequently observed.

As seen in Table 5, the mixed model analysis revealed significant effects of brain regions (Figure 4) and frequency bands on the observed coherence. Coherence however was not affected by the child's sex or residential condition at *p* < 0.05. In addition, there were significant two-way interactions on the observed coherence values: (1) child's sex and frequency band, (2) residential status and frequency band, and (3) brain region and frequency band. Inspecting the marginal means revealed that outpatient participants demonstrated higher coherence with their parents in the *α* band and female participants demonstrated lower coherence in the *α* band. Similarly, brain region and frequency band had significant effects on Granger influence (Figure 4). Furthermore, the direction of analysis (child to parent vs. parent to child) significantly affected Granger values. However, there was no significant effect of child's sex or residential status on Granger influence (*p* < 0.05). The interactions of (1) direction and frequency band, and (2) residential status and brain region exerted significant effects on Granger influences (Table 6).

**Table 5:** F-statistics and *p*-values from the linear mixed model of the effect of child's sex, residential status, brain region, frequency band, and interactions thereof, on the observed coherence measures.

**Effects**		***p*-value**
Sex	F(1, 6.4): 0.10	0.762
Residential status	*F*_(1,1.9)_: 3.13	0.228
Brain region	*F*_(15, 7088.2)_: 3.70	< 0.001
Frequency band	*F*_(4, 7088.2)_: 1989.25	< 0.001
Sex × Region	*F*_(15, 7088.2)_: 0.59	0.886
Sex × Frequency band	*F*_(4, 7088.2)_: 12.66	< 0.001
Residential status × Brain region	*F*_(15, 7088.2)_: 1.43	0.121
Residential status × Frequency band	*F*_(4, 7088.2)_: 55.04	< 0.001
Brain region × Frequency band	*F*_(60, 7088.2)_: 1.83	< 0.001

**Table 6:** F-statistics and *p*-values from the linear mixed model of the effect of direction of influence, child's sex, condition, brain region, frequency band, and interactions thereof, on the observed Granger influences.

**Effects**		***p*-value**
Direction	*F*_(1,14345.9)_: 19.42	< 0.001
Sex	*F*_(1, 5.8)_: 0.02	0.881
Residential status	*F*_(1, 1.1)_: 0.02	0.917
Brain region	*F*_(15, 14345.9)_: 23.59	< 0.001
Frequency band	*F*_(4, 14345.9)_: 283.58	< 0.001
Direction × Child's sex	*F*_(1, 14345.3)_: 0.62	0.43
Direction × Participant's condition	*F*_(1, 14345.9)_: 0.73	0.393
Direction × Brain region	*F*_(15, 14345.9)_: 0.01	0.998
Direction × Frequency band	*F*_(4, 14345.9)_: 7.11	< 0.001
Sex × Brain region	*F*_(15, 14345.9)_: 1.51	0.092
Sex × Frequency band	*F*_(4, 14345.9)_: 45.76	< 0.001
Residential status × Brain region	*F*_(15, 14345.2)_: 1.88	0.02
Residential status × Band	*F*_(4, 14345.9)_: 52.85	< 0.001
Brain region × Frequency band	*F*_(60, 14345.9)_: 6.24	< 0.001

## 4. Discussion

We documented contemporaneous brain signals and their directional influences in non-speaking children with severe disabilities and one of their cognate parents, during music therapy.

### 4.1. Synchronous Brain Regions

The observed interpersonal brain couplings were primarily concentrated in the prefrontal and frontal areas that are often implicated in empathic function (Shamay-Tsoory et al., 2003, 2009; Mitchell et al., 2005; Schulte-Rüther et al., 2007; Nummenmaa et al., 2008). The children were not able to see or hear their parents during the sessions. Thus, synchronized activation of empathy-related areas in both the children and parents suggest that the latter vicariously experienced the child-therapist interaction. In particular, there was significant coupling between Broca's area (parental brain), which is associated with emotional empathy (Schulte-Rüther et al., 2007; Nummenmaa et al., 2008) and dorsolateral prefrontal cortex (child's brain), which is linked to cognitive empathy (Shamay-Tsoory et al., 2003, 2009). This contralateral, regional interbrain coupling implies that while the children were cognitively engaged with their music therapist, the parents were emotionally attuned to their child's experience (Rankin et al., 2006; Decety and Ickes, 2009).

The ventrolateral frontal areas (including Broca's area) putatively support emotional empathy (Schulte-Rüther et al., 2007; Nummenmaa et al., 2008), a supposition corroborated by lesion studies where localized frontal damage leads to impaired emotional contagion (Shamay-Tsoory et al., 2009). The notion that the parents were attuned in an emotionally empathic manner to their children is further supported by the apparent association between Broca's area and the mirror neuron system (Fadiga et al., 2009), which is believed to be critical to social-cognitive processing (Schmidt, 2020).

The observation of synchronous involvement of the right frontal side of the children's brains may suggest invocation of non-verbal intuition, cognition, and creativity. Importantly, activity in the right prefrontal regions is affiliated with cognitive empathy, where one evaluates the mental states of others with reference to oneself (Shamay-Tsoory et al., 2003, 2009). Thus, the right prefrontal brain activity in the children might reflect some degree of allocentric perspective-taking, i.e., understanding of the therapist's thoughts, feelings and perceptions. In similar vein, the instances of significant ipsilateral (left-side) coupling between frontal areas of children and parents (at *β* and lower *γ* bands in the final sub-intervals of music therapy) suggest that both parents and children alike were harmonized with the emotional disposition of their counterpart (children and therapists, respectively), possibly as a consequence of observing facial expressions (Kesler-West et al., 2001) and affective prosody (Wildgruber et al., 2005).

### 4.2. Frequency Bands With Greatest Synchrony

Prefrontal and frontal activity in the *β* and *γ* bands is known to increase parametrically with working memory load (Howard et al., 2003; Spitzer et al., 2010; von Lautz et al., 2017). Thus, increased coupling in these bands might indicate synchronized active engagement in the music therapy sessions by both children and parents. Interestingly, we observed a paucity of *α* synchrony. This observation may in fact support the speculation that *β* and *γ* synchrony may have been indicative of empathic connection. In particular, Frenkel-Toledo et al. (2014) have reported associations between *μ* suppression (sensorimotor EEG signal power in the *α* frequency range) and mirror neuron system activation, which is putatively involved in cognitive empathy. Lübke et al. (2020) went further to report associations between *μ* suppression and a measure of state empathy while Babiloni et al. (2012) linked task-related *α* band power decrease to emotional empathy. Further, the lower *α* coherence in female participants during *post-hoc* analyses may suggest that the degree of empathic experiences varied depending on the child's sex. Indeed, many studies have reported generally heightened empathic traits in females (for an extensive review, see Christov-Moore et al., 2014): superior nonverbal emotion recognition (McClure, 2000; Schirmer et al., 2007), greater susceptibility to emotional contagion (Doherty et al., 1995; Magen and Konasewich, 2011), and advantages in mentalizing (perspective-taking) (Gardner et al., 2012).

### 4.3. Increase in Inter-brain Synchrony

Both coherence and Granger influence exhibited significant increases over the course of a 15-min music therapy session in frontal brain regions, in the *β* and low *γ* bands. In other words, the majority of parent and child dyads became more neurophysiologically "in sync" during a music therapy session. This is an interesting finding as it indicates that not only are the sample of non-speaking children with severe disabilities capable of physiologically influencing their parents (i.e., significant directional Granger values from child to parent), but that this influence is dynamic and labile. Indeed, studies of infants and mothers have reported that their interpersonal bio-behavioral synchrony can fluctuate with episodic gaze, vocalizations and touch (Feldman, 2012). Further, Davis et al. (2018) draw attention to proximal (such as the shared task at hand) and distal (such as family risk) contexts that may impact physiological synchrony. Our data would seem to suggest that a music therapy experience is a facilitatory context which can promote child-parent neural synchrony.

The parent-to-child Granger influence may appear odd at first glance, since the child was not able to see or interact with the parent. However, it is important to remember that the Granger measure in this case ascertains the contribution of previous values of the parent's brain signals to the prediction of current values of the child's brain signals. As such, the measure ought to be considered a quantification of the predictability of the child's signals on the basis of recent past values of the parent's signals. Alternatively, one might think of the Granger measure as capturing the strength with which the parent's signals lead the child's signals. In our study, it is indeed conceivable that the parent tended to anticipate the response of the child, given that the selected songs were familiar and personalized to each dyad. Recent literature has demonstrated that interpersonal autonomic synchrony between people may arise in the absence of face-to-face interaction, while jointly watching a positively or negatively valenced movie clip (Golland et al., 2015) or attending a live monolog performance (Ardizzi et al., 2020). In the former study, the degree of synchronization was further correlated with the level of convergence of subjective emotional experience. In terms of neural synchrony, Azhari et al. (2019) observed heightened prefrontal cortex hemodynamic signal alignment between spouses when attending to salient infant and adult vocalizations in each other's presence compared to experiencing the same alone or with a non-spousal control. Collectively, these studies suggest that "co-presence" (Golland et al., 2015) may be a critical contributor to physiological synchrony, ushering its emergence among people, without interpersonal communication, but simply through a joint sensory experience. In our study, the parent and child contemporaneously experienced, proximal to each other, salient musical stimuli presented by the therapist, without face-to-face interaction, and hence the observed parent-to-child synchrony may in part be attributable to their co-presence.

### 4.4. Music Therapy Context

The observed neurological correspondence between children and their parents in *β* and low *γ* bands may reflect neurophysiological responsiveness of the non-speaking children to the music therapy sessions. Indeed, Thompson and McFerran (2015) contend that music therapy "creates engaging and motivating conditions for interactions with others" and Mendelson et al. (2016) found that music therapy promoted verbal responsiveness in children with autism and other developmental disabilities.

Our findings suggest that music therapy seems to support the emergence of interbrain synchrony between parent and child. This may not be surprising given that one of the fundamental goals of music therapy is the establishing or re-establishing of interpersonal relationships (Meadows, 1997). Moreover, in a review of music therapy interventions with children with disabilities, Brown and Jellison (2012) reported that pediatric studies most often reported effective or partially effective treatment of social behaviors while Pavlicevic et al. (2014) found that music therapy afforded opportunities for developing and sustaining friendships in adults with disabilities. The latter study also lends credence to the choice of music therapy as a setting for studying interpersonal synchrony as families and young adults with severe learning disabilities ascribed relational and social values to long-term music therapy (Pavlicevic et al., 2014).

The interpretation that the observed spectral coherence may reflect mutual engagement may in part be supported by the fact that functional outcomes of music therapy often include children's ability to participate in turn-taking, maintain attention (Perry, 2003) and learn social reciprocity (Hussey et al., 2008). That music therapy affords a propitious setting for neural synchrony may be germane to the theoretical perspective that affective attunement between child and parent is musical and improvisational in nature (Lindstrøm et al., 2016).

### 4.5. Clinical Implications

The present study, while based on a modest sample, does have important implications for understanding the capacity of non-speaking children with severe disabilities for bio-behavioral neurophysiological synchrony. Our findings suggest that parent-child dyads can possess the ability to synchronize physiologically, in face of severe disability and without the need for making any vocal or gestural interactions. This tendency may be an instantiation of what Golland et al. (2015) termed "co-presence," where they found that the mere fact of being in the presence of another, in the absence of direct communication, can nonetheless lead to autonomic (heart rate and electrodermal activity) synchronization between people. While the participants in Golland et al. (2015) watched emotional movies, our findings suggest that music therapy with personalized content may also serve as a scaffold for physiological co-regulation.

The surprising finding that the outpatient dyads tended to exhibit stronger synchrony than their inpatient counterparts, albeit on the basis of small subsamples, supports the notion that the nature of familial relationships has a role in determining the levels of parent-child physiological co-regulation (Davis et al., 2018). Finally, our findings provide preliminary evidence that while caring for a child with severe disability is a known familial stressor (Burke et al., 2020), parent-child neurophysiological synchrony can be preserved and in fact, labile in a facilitatory, music therapy context. Future research may nonetheless consider documenting the mental health of caregivers as parental stress can diminish brain-to-brain synchrony between mothers and their young children (Azhari et al., 2019). Conversely, the presence of physiological synchrony may not always connote positive experiences and can in fact be maladaptive in response to negative interactions (Oshri et al., 2020).

### 4.6. Limitations

While we observed significant contralateral coupling of prefrontal and frontal brain regions, these regions are also critical to many other functional brain networks. For instance, Broca's area is commonly involved in language processing and one could surmise that coupling in this region could have simply indicated a synchronization of linguistic processing in response to music therapy. Therefore, we cannot definitively attribute the observed coherence and Granger influences to emotional empathy between parent and child. Future studies may yoke brain measurement with self-reported empathy scores throughout music therapy to shed light on the relationship between interbrain coupling and empathic function.

We did not control for time of day in our data collection but had to accommodate the schedules of our participants, many of whom had complex care needs. Nonetheless, diurnal rhythms can influence the level of physiological synchronization (Davis et al., 2018), and thus future studies may consider standardizing data collection times across participant where feasible.

Our participants ranged in developmental stage and clinical diagnoses. The propensity for synchrony with parents may depend on the developmental stage of the child (Harrist and Waugh, 2002) and in particular, their level of independence, e.g., more or less reliant on parental support, and time spent in co-habitation. Likewise, the lability of certain physiological responses and hence capacity for synchrony, may be blunted in certain clinical populations (Blain-Moraes and Chau, 2012). Future research may thus consider a narrower developmental and diagnostic sample.

A wealth of literature exists on rhythmic auditory stimulation and auditory-motor entrainment (Ghai et al., 2018). From our study data, we were not able to discriminate the contribution of synchrony due strictly to the rhythmic components of the music from that due to physical presence and shared emotional experiences. A future study may seek to quantify brain coupling due exclusively to listening to a common piece of music where dyadic participants are physically isolated from one another.

We did not have a balanced sample of males and females. From childhood through to adolescence, females are found to express more prosocial, sympathetic, and empathetic traits than males (Rose and Rudolph, 2006; Chaplin and Aldao, 2013). Future studies with larger, balanced samples of both males and females are required. Furthermore, both mothers and fathers of children should be recruited in future studies and parental demographic characteristics should be recorded to compare respective levels of neurophysiological coherence with their children and explore potential association with personal attributes. Gender-based differences in child-parent dyadic interactional synchrony have been previously noted (De Mendonça et al., 2019).

## 5. Conclusion

We observed significant neurophysiological synchrony as measured by coherence and Granger influence in a dyadic sample of non-speaking children with severe physical disabilities and cognate parents during music therapy sessions where the musical content was tailored to the child's preferences. Coherence was most prominent in contralateral frontal brain regions (child right and parent left) in the *β* and low *γ* bands. Further, both coherence and Granger influence increased significantly over the course of a music therapy session. Collectively, these findings suggest a cognitive-emotional alignment between child and parent that is responsive to music therapy. Further investigations on the facilitative effect of music therapy on interbrain synchrony and empathic connection between children and their familial caregivers are warranted.

## Data Availability Statement

The datasets presented in this article are not readily available because of ethical constraints. Requests to access the datasets should be directed to tchau@hollandbloorview.ca.

## Ethics Statement

The studies involving human participants were reviewed and approved by Holland Bloorview Kids Rehabilitation Hospital. Written informed consent to participate in this study was provided by the participants' legal guardian/next of kin.

## Author Contributions

SK and TC designed the study. SK collected the data. AS and SK performed the data analyses. JM and KK edited the manuscript and wrote parts of the discussion. TC, KK, and AS made substantive revisions to the manuscript. All authors contributed to the article and approved the submitted version.

## Conflict of Interest

The authors declare that the research was conducted in the absence of any commercial or financial relationships that could be construed as a potential conflict of interest.

## Footnotes

^1^www.emotiv.com
